# African ancestry is associated with cluster-based childhood asthma subphenotypes

**DOI:** 10.1186/s12920-018-0367-5

**Published:** 2018-05-31

**Authors:** Lili Ding, Dan Li, Michael Wathen, Mekibib Altaye, Tesfaye B. Mersha

**Affiliations:** 1 Division of Biostatistics and Epidemiology, Department of Pediatrics, Cincinnati Children’s Hospital Medical Center, Cincinnati, OH USA; 2 Alzheimer’s Therapeutic Research Institute, Keck School of Medicine, University of Southern California, San Diego, CA USA; 3 Division of Asthma Research, Department of Pediatrics, Cincinnati Children’s Hospital Medical Center, University of Cincinnati, 3333 Burnet Ave, Cincinnati, OH 45229 USA

**Keywords:** Childhood asthma, Cluster analysis, Genetic ancestry, Subphenotypes

## Abstract

**Background:**

Childhood asthma is a syndrome composed of heterogeneous phenotypes; furthermore, intrinsic biologic variation among racial/ethnic populations suggests possible genetic ancestry variation in childhood asthma. The objective of the study is to identify clinically homogeneous asthma subphenotypes in a diverse sample of asthmatic children and to assess subphenotype-specific genetic ancestry in African-American asthmatic children.

**Methods:**

A total of 1211 asthmatic children including 813 in the Childhood Asthma Management Program and 398 in the Childhood Asthma Research and Education program were studied. Unsupervised cluster analysis on clinical phenotypes was conducted to identify homogeneous subphenotypes. Subphenotype-specific genetic ancestry was estimated for 167 African-American asthmatic children. Genetic ancestry association with subphenotypes/clinical phenotypes were determined.

**Results:**

Three distinct subphenotypes were identified: a moderate atopic dermatitis (AD) group with negative skin prick test (SPT) and preserved lung function; a high AD group with positive SPT and airway hyperresponsiveness; and a low AD group with positive SPT and lower lung function. African ancestry at asthma genome-wide association study (GWAS) SNPs differed between subphenotypes (64, 89, and 94% for the three subphenotypes, respectively) and was inversely correlated with AD; each additional 10% increase in African ancestry was associated with 1.5 fold higher in IgE and 6.3 higher odds of positive SPT (all *p*-values < 0.0001).

**Conclusions:**

By conducting phenotype-based cluster analysis and assessing subphenotype-specific genetic ancestry, we were able to identify homogeneous subphenotypes for childhood asthma that showed significant variation in genetic ancestry of African-American asthmatic children. This finding demonstrates the utility of these complementary approaches to understand and refine childhood asthma subphenotypes and enable more targeted therapy.

**Electronic supplementary material:**

The online version of this article (10.1186/s12920-018-0367-5) contains supplementary material, which is available to authorized users.

## Background

Childhood asthma is a heterogeneous chronic airway disease with various clinical phenotypes [1, 2]. Its phenotypic and biologic heterogeneity contributes to the challenges clinicians face in its diagnosis and effective management [3]. It is therefore crucial to clearly define subphenotypes of asthma with homogeneous clinical characteristics in order to search for better asthma management and to develop novel therapeutic strategies. Although a large number of clinical phenotypes are often collected in childhood asthma studies, asthma genetic study has been mostly focused on case-control disease status. Such an endpoint-based analysis ignores the complexity of asthma phenotype [4–6]. In addition, although there is ample evidence for an intrinsic genetic variation among racial/ethnic populations [7, 8] suggesting possible genetic ancestry variation in childhood asthma, most genetic analyses rely on self-reported race thus do not account for the potential contribution of genetic ancestry to disease variation in diverse populations.

An approach to overcome the phenotypic heterogeneity of childhood asthma is to identify homogeneous subgroups by establishing either classical “endotype”, based on experts’ criteria, or statistical phenotype clustering on asthma clinical phenotypes. The latter has been successfully applied to identify clinically relevant subgroups of asthmatics and other airway diseases [9–17]. However, these studies differ in some key elements: variation in phenotyping, analytical approaches used and the patient population under study. These differences limit the comparability of the identified subphenotypes and pose difficulty in applying clustering results to individual patients. Furthermore, little is understood regarding the genetic ancestry of the identified subphenotypes.

The objective of the study is to investigate childhood asthma phenotypic heterogeneity and genetic ancestry variations and their relationships. Specifically, we used childhood asthma data from the NIH controlled database of Genotype and Phenotype (dbGaP) to identify homogeneous subphenotypes, determine clinical phenotypes, estimate subphenotype-specific genetic ancestry, and analyze the relationship between ancestry and subphenotypes using a stepwise approach incorporating cluster analysis, classification tree analysis, and genetic ancestry analyses [9–16, 18, 19]. Our goal is to combine both cluster and genetic ancestry to identify biologically-relevant subphenotypes in childhood asthma.

## Methods

### Data

The database of Genotypes and Phenotypes (dbGaP) is the repository for both genotype and phenotype data from most NIH-funded GWAS and other whole-genome or exome sequence data. We used baseline data from the SNP Health Association Resource (SHARe) Asthma Resource Project (SHARP) (phs000166.v2.p1), the National Heart, Lung, and Blood Institute’s clinical research trials on asthma, specifically, the Childhood Asthma Management Program (CAMP) and the Childhood Asthma Research and Education (CARE) network. The CAMP is a multicenter, randomized, double-masked clinical trial designed to determine the long-term effects of three inhaled treatments for mild to moderate childhood asthma [20]. The CARE data evaluates current and novel therapies and management strategies for children with asthma. Individual level data with asthma diagnosis is available for 1211 subjects through Authorized Access, including 813 in CAMP and 398 in CARE.

An array of phenotypic variables have been harmonized across the CAMP and CARE datasets, including demographics and participant characteristics; intermediate asthma phenotypes such as lung function, skin prick test (SPT), serum total immunoglobulin (IgE), and atopic dermatitis (AD), as well as environmental exposure. See Table 1 for a complete list of variables.

**Table 1:** Demographic, clinical phenotypes and environmental exposures of CAMP and CARE study participants

	CAMP (*N* = 813)	CARE(*N* = 398)	*p*-value
Age, Mean (SD), years	8.9 (2.1)	10.6 (2.8)	< 0.0001
Gender, No. (%)			0.8152
Male	500 (61.5)	242 (60.8)	
Female	313 (38.5)	156 (39.2)	
Race, No. (%)			< 0.0001
Caucasian	557 (68.5)	215 (54)	
African American	107 (13.2)	70 (17.6)	
Hispanic	77 (9.5)	78 (19.6)	
Other	72 (8.9)	35 (8.8)	
BMIZ at baseline, Mean (SD)	0.5 (1.0)	0.8 (1.0)	< 0.0001
Age of onset^a^, Mean (SD), years	3.0 (2.4)	3.7 (3.3)	< 0.0001
FEV1 PC20 meth^b^, Mean (SD), mg/ml	2.0 (2.4)	2.2 (3.1)	0.3602
FEV1 percent predicted^c^, Mean (SD)	93.4 (14.1)	97.1 (12.8)	< 0.0001
FVC percent predicted^d^, Mean (SD)	103.7 (13.1)	106.7 (12.2)	0.0002
FEV1/FVC ratio^e^, Mean (SD)	79.6 (8.3)	80.1 (8.0)	0.2937
Bronchodilator percent change^f^, Mean (SD)	10.7 (9.9)	9.4 (8.4)	0.0236
Blood eosinophils, Mean (SD), mm^3^	485.7 (409.2)	408.8 (319.5)	0.0011
IgE, Mean (SD), ng/ml	1129.8 (2081.9)	330.6 (445.4)	< 0.0001
Average AM peak flow^g^, Mean (SD), L/min	250.9 (64.4)	271.1 (92.4)	< 0.0001
Average AM symptoms^h^, Mean (SD)	0.61 (0.45)	0.51 (0.40)	< 0.0001
Environmental smoke^i^, No. (%)	339 (41.7)	166 (41.7)	0.0256
In utero smoke^j^, No. (%)	107 (13.2)	54 (13.6)	0.8060
Atopic dermatitis^k^, No. (%)	199 (24.4)	155 (38.9)	< 0.0001
One or more positive SPT^l^, No. (%)	716 (88.1)	312 (78.4)	0.0002

^a^Age at first asthma symptoms

^b^The dose of methacholine that is required to decrease FEV1 by 20%

^c^Forced expiratory volume, the maximal amount of air one can forcefully exhale in one second converted to a percentage of normal based on one’s height, weight, body composition, and race

^d^Forced vital capacity, the amount of air a person can expire after a maximum inspiration second converted to a percentage of normal based on one’s height, weight, body composition, and race

^e^Also called Tiffeneau-Pinelli index, is a calculated ratio used in the diagnosis of obstructive and restrictive lung disease. It represents the proportion of a person’s vital capacity that they are able to expire in the first second of expiration

^f^Post bronchodilator percent change from baseline: 100*(POSFEV - PREFEV)/PREFEV

^g^The maximum flow rate generated during a forceful exhalation, starting from full lung inflation; average of daily measurements up to 4 weeks prior to visit with a minimum of 7 days, recorded in daily diary card

^h^Maximum of daily wheezing and coughing then average of daily measurements up to 4 weeks prior to visit with a minimum of 7 days, recorded in daily diary card

^i^Either parent smoked during trial or home exposure to smoke prior to trial enrollment

^j^Mother smoked when pregnant with participant

^k^Child had atopic dermatitis for 2 years and was seen by a doctor for it

^l^One or more skin prick test positive

We downloaded CAMP and CARE genotype data which were performed using 1 million single nucleotide polymorphisms (SNPs) in the Affymetrix 6.0 chip and stored in the database of dbGaP (accession number phs000166.v2.p1). Quality control criteria included minor allele frequency ≥ 0.05, Hardy-Weinberg equilibrium (*p* ≥ 10^− 5^), ≤ 5% missing rate per person, ≤ 5% missing rate per SNP, families with less than 5% Mendel errors and SNPs with less than 10% Mendel error rate [21].

### Hierarchical cluster analysis (HCA)

*HCA* is a hypothesis free statistical method to group subjects into relatively homogeneous sub-clusters according to similarity quantification based on a set of critical characteristic variables. The grouping is constructed such that the similarity is strong between members of the same cluster and weak between members of different clusters. The baseline phenotypic measures listed in Table 1 were included in the cluster analysis. To reduce collinearity, we examined the variables for absolute correlation (> 0.80). We also assessed missing pattern of the phenotypes and planned to exclude measures with ≥10% missingness from the analysis. Blood eosinophils (EOS) and IgE were log transformed.

Since we have mixed types of variables, i.e., continuous and categorical, Gower’s distance [22] was used as a similarity index. To avoid inconsistent cluster solutions due to changes in scale of the variables and heavy impact of variables with larger standard deviations, Gower’s standardization, based on the range, was applied. HCA was then carried out with Ward’s minimum-variance method [23]. Consensus between a pseudo *F* and a pseudo *t*^2^ statistics [24, 25] was used to select the number of clusters. The number of clusters was also guided by clinical characteristics in addition to statistical considerations.

Descriptive statistics of all variables were obtained and compared across clusters using analysis of variance, Kruskal-Wallis, or Chi-square tests as appropriate. Conditional inference trees [26], a non-parametric class of regression trees that embeds tree structured regression models into a well-defined theory of conditional inference procedures, was used to identify intermediate phenotypes that distinguish the subphenotypes. The cluster analysis was first carried out on the CAMP data and repeated on the CARE data. Replication of the clustering results was examined between the two studies as well as with previously published studies.

Additional analyses were run to investigate if the subphenotypes were associated with clinical outcomes. Two clinical outcomes were examined, number of prednisone bursts (an anti-inflammatory oral steroid medication) since last visit, and number of ER visit or hospitalizations since last visit. Number of prednisone bursts since last visit was modeled as a count variable using Poisson regression with a random subject effect. Number of ER visit or hospitalizations since last visit was dichotomized (given over 95% of the subjects did not had an ER visit or hospitalization), and modeled using a logistic regression with a random subject effect. Potential covariates included age, sex, race, visit month, time since last visit, treatment, and subphenotypes that were significantly associated with the outcome (adjusted *p*-value < 0.05). All analyses were run for CAMP and CARE data separately. All the above analyses were conducted in SAS version 9.3 (SAS Institute Inc., Cary, NC, USA) and R [27].

### Genetic ancestry analysis

Genetic ancestry was estimated using both genome-wide SNPs and asthma-specific GWAS SNPs for African-American asthmatic individuals in CAMP and CARE. Supervised approach in the ADMIXTURE software program [28] was use to estimated global genetic ancestry, where SNP data of 108 YRI (Yoruba in Ibadan, Nigeria) and 99 CEU (Utah Residents (CEPH) with Northern and Western Ancestry) individuals from the 1000 Genomes Project were included as surrogates for European and African ancestry. The reference populations and the CAMP/CARE subjects shared 857,127 genetic markers across all autosomes, which reduced to 225,374 SNPs after linkage disequilibrium (LD) pruning with window of 50 (kb), 10 kb window shift and a r2 value of 0.2.

Asthma GWAS SNPs, 157 in total, were retrieved from the GWAS catalog [29] and STRUCTURE software [30] was used to estimate African ancestry proportion at asthma GWAS SNPs. CEU and YRI individuals from the 1000 Genomes Project were used as parental populations.

Correlations between genetic ancestry and the subphenotypes derived by clustering and the discriminate factors of the subphenotypes were examined using the Kruskal-Wallis test, Wilcoxon rank-sum test, Spearman correlation coefficient, or linear regression as appropriate.

## Results

Participants from CAMP and CARE were different except in sex, exposure to in utero smoking, PC20, and FEV1/FVC ratio (Table 1). All pairwise Spearman correlation coefficients were less than 0.60, except between FEV1 percent predicted and FVC percent predicted (0.71) and between FEV1/FVC and maximum bronchodilator percent change (− 0.65). No variables had more than 10% of missing values.

### HCA identified distinct subphenotypes

#### Clustering on CAMP cohort identified distinct subphenotypes

Three clusters were identified from CAMP data (Table 2). Members of cluster 1 had a moderate AD rate (15.3%) and all but one had negative SPT (99%). This group also had the lowest age at baseline, age at onset of asthma, bronchodilator percent change, EOS, IgE level, AM peak flow, and AM symptoms, and highest body mass index z-sore (BMIZ), PC20, FEV1 percent predicted, and FEV1/FVC ratio. All these characteristics, but BMIZ and AM symptoms, were statistically different across the clusters at a significant level of 0.05. This is the moderate AD group with negative SPT and preserved lung function.

**Table 2:** CAMP hierarchical clustering results

	Cluster 1 (*N* = 98)	Cluster 2 (*N* = 171)	Cluster 3 (*N* = 544)	*p*-value
Age (years)	7.8 (1.9)	8.7 (2.1)	9.2 (2.1)	< 0.0001
Gender No. (%)				0.0675
Male	50 (51.0)	105 (61.4)	345 (63.4)	
Female	48 (49.0)	66 (38.6)	199 (36.6)	
Race No. (%)				0.0153
Caucasian	82 (83.7)	116 (67.8)	359 (66.0)	
African American	9 (9.2)	25 (14.6)	73 (13.4)	
Hispanic	5 (5.1)	12 (7.0)	60 (11.0)	
Other	2 (2.0)	18 (10.5)	52 (9.6)	
BMIZ	0.7 (1.0)	0.6 (1.1)	0.5 (1.0)	0.0929
Age of onset (years)	2.4 (2.2)	2.8 (2.2)	3.2 (2.5)	0.0017
FEV1 PC20 meth (mg/ml)	2.9 (2.8)	1.8 (2.2)	2.0 (2.4)	0.0005
FEV1 percent predicted	96.3 (14.5)	95.0 (14.5)	92.4 (13.9)	0.0117
FVC percent predicted	103.5 (14.3)	104.1 (13.6)	103.6 (12.7)	0.895
FEV1/FVC ratio	82.9 (7.0)	80.6 (8.2)	78.7 (8.3)	< 0.0001
Bronchodilator percent change	7.3 (6.9)	11.4 (10.1)	11.1 (10.1)	0.0012
Blood eosinophils (mm^3^)	228.9 (197.9)	579.7 (442.4)	504 (408.8)	< 0.0001
IgE (ng/ml)	200.5 (449.1)	1579 (2624.2)	1161 (2022.7)	< 0.0001
Average AM peak flow (L/min)	230.9 (55)	249.8 (67.5)	254.8 (64.4)	0.0040
Average AM symptoms	0.52 (0.40)	0.61 (0.46)	0.63 (0.45)	0.100
Environmental smoke No. (%)	42 (42.9)	60 (35.1)	237 (44.1)	0.1291
In utero smoke No. (%)	19 (19.4)	11 (6.4)	77 (14.2)	0.0046
Atopic dermatitis No. (%)	15 (15.3)	167 (97.7)	17 (3.1)	< 0.0001
Positive SPT No. (%)	1 (1)	171 (100)	544 (100)	< 0.0001

Mean and SD for continuous variables and No. (%) for categorical variables

Members of cluster 2 had a high rate of AD (97.7%) and all had one or more positive SPT. This group also had the highest EOS and IgE level, and lowest bronchodilator percent change among the 3 clusters. This is the high AD group with positive SPT and airway hyperresponsiveness.

Members of cluster 3 had the highest age at baseline and age onset of asthma and lowest BMIZ. This group had also the lowest FEV1 percent predicted and FEV1/FVC ratio, and highest AM symptoms. Furthermore, members of cluster 3 were mostly AD free and all had one or more positive SPT, moderate EOS and IgE levels, but lower lung function measures and higher AM symptoms compared to the other clusters. This is the low AD group with positive SPT and lower lung function.

#### Clustering on CARE cohort replicated the subphenotypes identified in CAMP

Three clusters were identified in CARE (Table 3). Members of cluster 1 had a moderate rate of AD (35%) and none of them had a positive SPT. This group also had the lowest bronchodilator percent change, EOS, IgE, AM peak flow, and lowest AM symptoms. All these characteristics, but the last, were statistically different across the clusters at a significant level of 0.05. This is the moderate AD group with negative SPT and preserved lung function similarity identified in CAMP.

Members of cluster 2 had a high rate of AD (98.4%) and one or more positive SPT (95.3%). This group also had the highest EOS and IgE level among the 3 clusters. This is the high AD asthma group with positive SPT and airway hyperresponsiveness similarly identified in CAMP.

Members of cluster 3 had the highest age at baseline and age onset of asthma, were mostly AD free (3.3%) and all had one or more positive SPT (92.2%), had moderate EOS and IgE levels, but higher AM symptoms compared to the other clusters. This is the low AD group with positive SPT and lower lung function similarly identified in CAMP.

**Table 3:** CARE hierarchical clustering results

	Cluster 1 (*N* = 60)	Cluster 2 (*N* = 129)	Cluster 3 (*N* = 209)	*p*-value
Age (years)	10.1 (2.4)	10.1 (2.5)	11.0 (3.1)	0.0124
Gender No. (%)				0.1185
Male	30 (50)	77 (59.7)	135 (64.6)	
Female	30 (50)	52 (40.3)	74 (35.4)	
Race No. (%)				0.4519
Caucasian	40 (66.7)	67 (51.9)	108 (51.7)	
African American	8 (13.3)	24 (18.6)	38 (18.2)	
Hispanic	8 (13.3)	24 (18.6)	46 (22.0)	
Other	4 (6.7)	14 (10.9)	17 (8.1)	
BMIZ	0.9 (0.9)	0.8 (1.0)	0.8 (1.0)	0.5920
Age of onset (years)	3.6 (3.5)	3.1 (2.6)	4.1 (3.5)	0.0215
FEV1 PC20 meth (mg/ml)	3.3 (3.3)	1.6 (2.4)	2.3 (3.4)	0.0031
FEV1 percent predicted	97.2 (13.4)	96.3 (13.1)	97.6 (12.5)	0.655
FVC percent predicted	104.7 (10.7)	107.2 (12.5)	106.9 (12.3)	0.378
FEV1/FVC ratio	81.6 (8.5)	79.0 (8.0)	80.4 (7.9)	0.101
Bronchodilator percent change	6.7 (7.4)	9.9 (7.4)	9.8 (9.0)	0.0271
Blood eosinophils (mm^3^)	245.7 (211.5)	444.4 (322.1)	435.0 (330.2)	< 0.0001
IgE (ng/ml)	63.5 (133.9)	424.5 (537.1)	347.4 (430.1)	< 0.0001
Average AM peak flow (L/min)	255.4 (68.7)	258.6 (81.0)	283.3 (102.3)	0.0209
Average AM symptoms	0.43 (0.32)	0.50 (0.40)	0.53 (0.42)	0.202
Environmental smoke No. (%)	28 (46.7)	62 (48.1)	104 (49.8)	0.8985
In utero smoke No. (%)	1 (1.7)	18 (14.0)	35 (16.9)	0.0121
Atopic dermatitis No. (%)	21 (35)	127 (98.4)	7 (3.3)	< 0.0001
Positive SPT No. (%)	0 (0)	123 (95.3)	189 (92.2)	< 0.0001

Mean and SD for continuous variables and No. (%) for categorical variables

### Atopic dermatitis status and SPT distinguished the subphenotypes

**Fig. 1:**
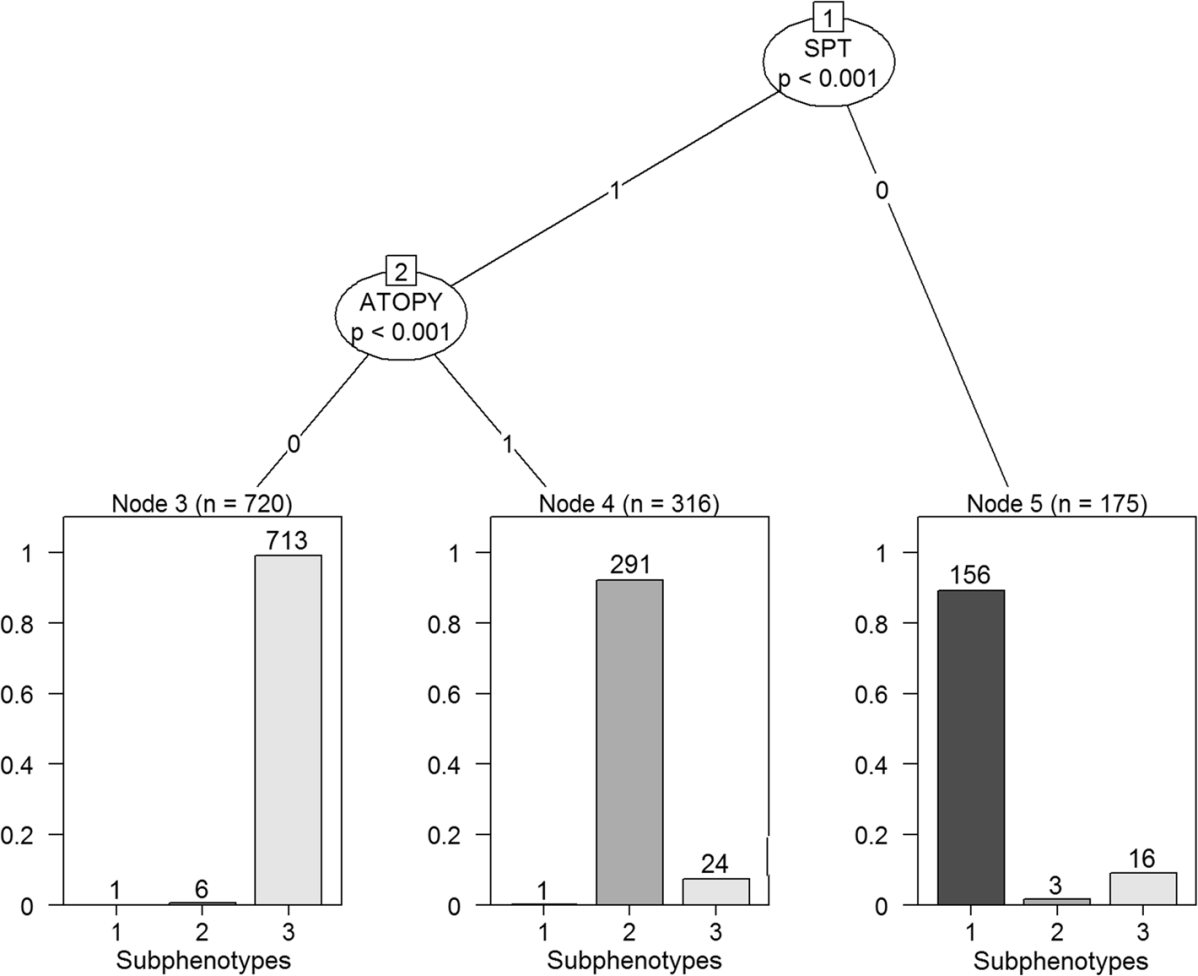
Conditional inference tree analysis of the three subphenotypes. SPT and atopic dermatitis are the top two factors distinguishing the subphenotypes. The prediction accuracy is 95.8%

Conditional inference trees analysis revealed that, in both CAMP and CARE data, AD and one or more positive SPT were the top two variables that best discriminated the individuals into the subphenotypes (Fig. 1, prediction accuracy 95.8%). Given the consistent findings across CAMP and CARE data, we combined the two datasets and grouped the three clusters individually identified in CAMP and CARE into three subphenotypes. One subphenotype was the moderate AD group with negative SPT and preserved lung function (subphenotype 1, *n* = 158), one was the high AD group with positive SPT and airway hyperresponsiveness (subphenotype 2, *n* = 300), and one was the low AD group with positive SPT and lower lung function (subphenotype 3, *n* = 753).

### Subphenotypes were associated clinical outcomes

Table 4 shows the association between the subphenotypes and clinical outcomes. In CAMP data, the incident rate of prednisone bursts since last visit for subphenotype 2 is 2.63 (1.45, 2.70) times the incident rate for subphenotype 1, and the incident rate of prednisone bursts since last visit for subphenotype 3 is 2.04 (1.56, 2.70) times the incident rate for subphenotype 1. Also in CAMP data, the odds of any ER visit or hospitalizations since last visit for subphenotype 3 is 1.54 (1.01, 2.23) times the odds for subphenotype 1. For CARE data, the odds of any ER visit or hospitalizations since last visit for subphenotype 2 is 0.32 (0.13, 0.98) times the odds for subphenotype 1, and the odds of any ER visit or hospitalizations since last visit for subphenotype 3 is 3.45 (1.47, 7.69) times the odds for subphenotype 2.

**Table 4:** Association between subphenotypes and number of prednisone bursts and any ER visit or hospitalizations since last visit

Number of prednisone bursts since last visit					
CAMP					
Predicted number of event			Incident rate ratios		
Subphenotype	Estimate (95% CI)	*p*-value	Subphenotypes	IRR (95% CI)	*p*-value
1	0.10 (0.08, 0.13)	< 0.0001	2 vs. 1	2.63 (1.45, 2.70)	< 0.0001
2	0.20 (0.16, 0.24)		3 vs. 1	2.04 (1.56, 2.70)	< 0.0001
3	0.20 (0.18, 0.22)		3 vs. 2	1.02 (0.83, 1.27)	0.8153
CARE					
Predicted number of event			Incident rate ratios		
Subphenotype	Estimate (95% CI)	*p*-value	Subphenotypes	IRR (95% CI)	*p*-value
1	0.08 (0.05, 0.14)	0.3534	2 vs. 1	0.93 (0.57, 1.54)	0.7880
2	0.08 (0.05, 0.12)		3 vs. 1	1.19 (0.76, 1.89)	0.4420
3	0.10 (0.07, 0.14)		3 vs. 2	1.28 (0.90, 1.82)	0.1666
Any ER visit or hospitalizations since last visit					
CAMP					
Predicted probability			Odds ratios		
Subphenotype	Estimate (95% CI)	*p*-value	Subphenotypes	OR (95% CI)	*p*-value
1	0.03 (0.02, 0.04)	0.1232	2 vs. 1	1.52 (0.95, 2.44)	0.0776
2	0.04 (0.03, 0.05)		3 vs. 1	1.54 (1.01, 2.33)	0.0434
3	0.04 (0.03, 0.04)		3 vs. 2	1.01 (0.75, 1.37)	0.9474
CARE					
Predicted probability			Odds ratios		
Subphenotype	Estimate (95% CI)	*p*-value	Subphenotypes	OR (95% CI)	*p*-value
1	0.02 (0.01, 0.05)	0.0155	2 vs. 1	0.35 (0.13, 0.98)	0.0458
2	0.01 (0.004, 0.02)		3 vs. 1	1.20 (0.56, 2.63)	0.6296
3	0.03 (0.02, 0.04)		3 vs. 2	3.45 (1.47, 7.69)	0.0039

### Genetic ancestry proportion varied at asthma GWAS SNPs among asthma subphenotypes

**Fig. 2:**
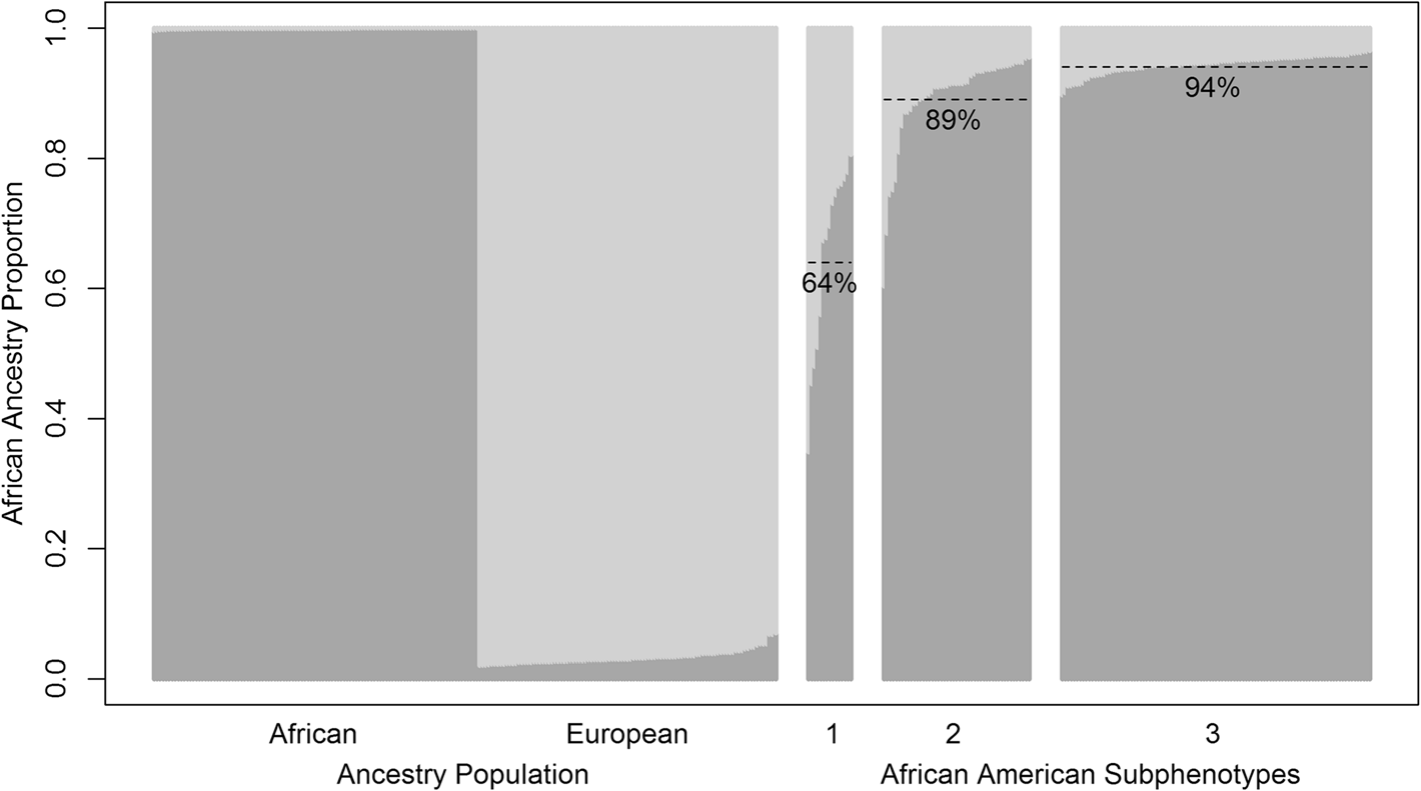
Population ancestry estimates of African American asthmatic individuals in CAMP and CARE at asthma GWAS SNPs by subphenotypes. Dashed lines indicate average proportions of African ancestry proportion at the asthma GWAS SNPs. Ibadan, Nigeria (YRI) and northern and western European (CEU) from the 1000 Genomes project were used as parental populations

**Fig. 3:**
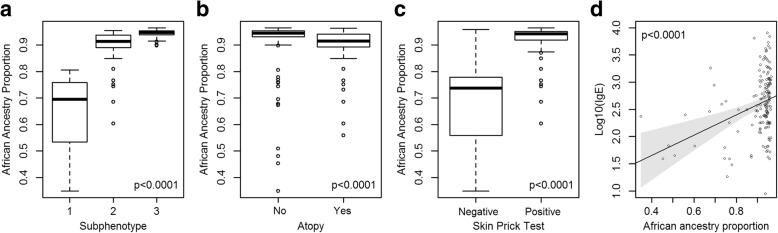
Boxplots and scatterplot of proportions of African ancestry at the asthma GWAS SNPs by: **a** subphenotypes, **b** Atopic dermatitis status, **c** SPT, and **d** IgE levels

The three subphenotypes had 15, 49, and 103 African American individuals, respectively. Global African ancestry proportion varies from 71.2 to 100% with mean 96.6% and standard deviation (SD) 7.2%. Higher global African ancestry was associated with AD (mean ± SD of African origin is 0.96 ± 0.08 for AD free vs. 0.98 ± 0.06 for AD subjects, *p*-value = 0.0294), but not with other clinical phenotypes. Proportion of African ancestry at asthma GWAS SNPs was correlated with the subphenotypes (mean 64.9, 89.4 and 94.4% for subphenotypes 1, 2, and 3, respectively, *p*-value < 0.0001, Figs. 2 and 3(a)). The subphenotypes were associated with lung function: FEV1 percent predicted is 96.8 ± 14.1, 95.3 ± 13.9, and 93.9 ± 13.7 (*p*-value = 0.0083); and FEV1/FVC ratio is 81.9 ± 7.6, 80.5 ± 8.1, and 79.0 ± 8.2 (*p*-value < 0.0001) for subphenotypes 1, 2, and 3, respectively. Furthermore, African ancestry at asthma GWAS SNPs was inversely associated with AD (median 0.95 with IQR (0.93, 0.95) for AD free vs. 0.92 (0.89, 0.94) for AD subjects, *p*-value < 0.0001, Fig. 3(b)). Additionally, genetic ancestry at asthma GWAS SNPs was associated with positive SPT with median and interquartile range (IQR) 0.94 (0.92, 0.95) for positive SPT individuals vs. 0.74 with IQR (0.59, 0.78) for negative SPT individuals (*p*-value < 0.0001, Fig. 3(c)). The odds of one or more positive SPT was 6.3 higher (95% confidence interval: (3.4, 13.8), *p*-value < 0.0001) with each additional 10% of African origin at asthma GWAS SNPs. African origin at asthma GWAS SNPs was also associated with IgE levels (Spearman correlation coefficient = 0.27, *p*-value = 0.0004) and IgE was 1.5 fold higher with each additional 10% of African origin (Fig. 3(d)).

## Discussion

Current clinical practice in childhood asthma treatment tends to use average patient care strategies. Such a “one size fits all” treatment approach faces major challenges when it is becoming clearer that childhood asthma is heterogeneous in pathogenesis. Our unbiased cluster and genetic ancestry analyses pointed toward three distinct phenotypic clusters with differences in clinical characteristics, genetic ancestry, and clinical outcomes, underscoring the clinical and genetic heterogeneity of asthma [10, 13, 17, 31]. Previous studies have also identified clusters with atopic or non-atopic asthma, clusters with preserved or lower lung function, and clusters with mild asthma [13, 14, 32]. It is reassuring that the two independent studies replicated the clustering results and there are similarities with previous clustering-based childhood asthma subphenotypes.

We determined genetic ancestry [33] using genome-wide SNPs and asthma GWAS SNPs for African-American asthmatic individuals in CAMP and CARE data. Our estimate of African global ancestry in asthmatic children is higher than what has been reported in different general populations confirming the higher prevalence of asthma in individuals with higher African ancestry than others. Our results showed that genetic ancestry at asthma GWAS SNPs differed between the childhood asthma subphenotypes and was associated with lung function, SPT, IgE levels, and AD. Previous studies have also showed association between genetic ancestry and asthma prevalence and related clinical phenotypes [34–42]. To our best knowledge, our study is the first to show the association between genetic ancestry at asthma GWAS SNPs and cluster-based subphenotypes in childhood asthma. Leveraging ancestry and cluster analyses to derive genetic and phenotypic homogeneity subgroups in childhood asthma demonstrates the utility of these approaches to characterize and understand the complexity of asthma towards individual based precision medicine strategies.

This study demonstrates that genetic ancestry at asthma GWAS SNPs is more strongly associated with asthma subgroups sharing similar clinical characteristics compared to broadly defined asthma. The results suggest that validation of genetic studies are more likely to be successful for replication studies carried-out in more homogeneous asthma cohorts (sharing similar clinical characteristics) compared to the multifactorial case-control status. In addition, the results indicate that ancestry-specific genetic loci of asthma are likely to be found by focusing on better defined asthma patients. Furthermore, genetic ancestry analysis in homogeneous asthma subgroups is suitable to refine the biological role of asthma susceptibility variants from GWAS studies in a given phenotype. For example, SNPs at *STARD3/PGAP3* are strongly associated with the high atopic dermatitis subgroup suggesting that *STARD3/PGAP3* may act on the allergic component of asthma [43]. Another example is that *ORMDL3/17q* locus is associated with asthma in multiple studies in the European ancestry but not in African ancestry asthmatic individuals [44]. We also investigated associations between asthma GWAS SNPs with the identified subphenotypes in CAMP and CARE data (methods and results in Additional file 1: Table S1). Several significant associations were identified at *p* = 0.05, but none after multiplicity adjustment, possibly due to small sample size and limited statistical power.

Our study had several limitations. First, participants in CAMP and CARE represent studies of childhood asthma, thus the results herein may not be applicable to adulthood asthma. Second, although we identified clinically relevant subphenotypes of childhood asthma using clinical phenotypes [45], the integration of this result with molecular and physiologic phenotyping may help to better understand childhood asthma pathogenesis for possibly more personalized therapeutic strategies. Furthermore, subgroup analyses of asthma may limit sample sizes and impair statistical power. However, given asthma is a highly heterogeneous phenotype, studying homogeneous subgroups of asthma patients not only recovers power limitation, but achieves more statistically significant results. Classifying asthma patients in more homogenous groups may help us to identify new susceptibility or modifying subphenotype-specific genes. Our ability to better define subtypes might help to predict who may respond to treatment vs subjects who may not. Future studies need to elucidate the mechanisms that distinguish each ancestral and clinical clusters to facilitate the development of targeted therapies and providing personalized treatments.

The present study has notable strengths. First, we were able to dissect childhood asthma heterogeneity into subphenotypes using cluster analysis of clinical phenotypes in one study and replicate the findings in an independent study. Second, we were able to show associations between the identified subphenotypes with asthma clinical outcomes. Third, analysis of genetic ancestry at asthma GWAS SNPs in childhood asthma clinical phenotypes provide biologically relevant subphenotype-specific results. Lastly, our study used a more accurate and direct assessment of genetic ancestry instead of self-reported race to determine the relationship between ancestry and childhood asthma subphenotypes and relevant clinical phenotypes. Studies have shown that people with the same self-reported race could have drastically different levels of genetic ancestry, and self-reported race may not be as accurate as direct assessment of genetic ancestry in predicting treatment outcomes [33]. Future studies to identify genetic ancestry-specific variants associated with a specific subphenotype are important as we move towards applying precision medicine paradigm. The finding indicates that African genetic ancestry at asthma GWAS SNPs are differentially associated with the asthma clinical subphenotypes. Unraveling the reasons why individuals with higher African origin at asthma GWAS SNPs had higher IgE level or rate of positive SPT is necessary to determine the potential clinical applications of our findings. In addition, genetic analysis based on more refined phenotypes may increase the statistical power and allow for the detection of population structure-specific phenotype-genotype associations that are undetectable otherwise.

## Conclusions

In conclusion, through our systematic clinical phenotype analysis, we identified distinct subphenotypes for childhood asthma using cluster analysis. Further genetic ancestry analysis showed correlations between African ancestry at asthma GWAS SNPs and childhood asthma subphenotypes and related clinical outcomes. Our results demonstrated that cluster analyses on clinical phenotypes followed by ancestry analysis can enhance the understanding of the phenotypic and genetic heterogeneity of childhood asthma. Our approach is distinct from previous efforts in that we developed cluster-based subphenotype and applied genetic ancestry analysis to define subphenotype-ancestry relationships which can be subsequently used as the basis of genetic ancestry based clinical risk prediction. Our findings suggest that defining asthma heterogeneous subgroups on the basis of clinical phenotypes and genetic ancestry proportion is an essential step to understand and refine patient subsets and enable more targeted therapy.

## Additional file

Additional file 1: **Table S1.** Association between asthma GWAS SNPs and subphenotypes. This file contains association results between asthma GWAS SNPs with the identified subphenotypes in CAMP and CARE data. (DOCX 24 kb)

## Abbreviations

AD: Atopic dermatitis
BMIZ: Body mass index z-sore
CAMP: the Childhood Asthma Management Program
CARE: the Childhood Asthma Research and Education network
CEU: Utah Residents with Northern and Western Ancestry
dbGaP: The database of Genotypes and Phenotypes
EOS: Blood eosinophils
FEV1: Forced expiratory volume, the maximal amount of air one can forcefully exhale in 1 s converted to a percentage of normal based on one’s height, weight, body composition, and race
FVC: Forced vital capacity, the amount of air a person can expire after a maximum inspiration second converted to a percentage of normal based on one’s height, weight, body composition, and race
GWAS: Genome-wide association study
HCA: Hierarchical cluster analysis
IgE: Serum total immunoglobulin
IQR: Interquartile range
LD: Linkage disequilibrium
PC20: The dose of methacholine that is required to decrease FEV1 by 20%
SD: Standard deviation
SHARe: The SNP Health Association Resource
SHARP: The SNP Health Association Resource Asthma Resource Project
SNP: Single nucleotide polymorphism
SPT: Skin prick test
YRI: Yoruba in Ibadan, Nigeria
