# Supplementary material for: African ancestry is associated with cluster-based childhood asthma subphenotypes
Source: BMC Med Genomics. 2018 May 31;11:51. doi: 10.1186/s12920-018-0367-5 (PMC5984446; doi:10.1186/s12920-018-0367-5)
Supplement: Supplementary file 1 — Table S1. Association between asthma GWAS SNPs and subphenotypes. This file contains association results between asthma GWAS SNPs with the identified subphenotypes in CAMP and CARE data. (DOCX 24 kb) [file 12920_2018_367_MOESM1_ESM.docx]

**Additional file**

**Associations between asthma GWAS SNPs and subphenotypes for Caucasian, African-American, and Hispanics**

We examined the association between 43 asthma GWAS SNPs shared between CAMP and CARE data and the subphenotypes for Caucasian, African-American, and Hispanic asthmatic children separately. No statistically significant associations were found after Bonferroni correction for multiple comparisons. Chi-square test results indicate association (p<0.05) between rs10197862, rs987870, rs1425902, and rs1930336 with the subphenotypes in Caucasians, rs7130588 and rs4815617 with the subphenotypes in African-Americans, and rs16937883, rs9319321, and rs10411428 with the subphenotypes in Hispanics. Results from Spearman correlation indicate nonzero correlation (p<0.05) between rs1837253, rs2378383, and rs11835157 with the subphenotypes in Caucasians, rs7775228 with the subphenotypes in African-Americans, and rs10411428 with the subphenotypes in Hispanics. Results are presented in Table S1 below.

Table S1. Association between asthma GWAS SNPs and subphenotypes

| **SNP** | **Chr** | **Related Genes** [1] | **Caucasian** | | | **African American** | | | **Hispanic** | | |
| --- | --- | --- | --- | --- | --- | --- | --- | --- | --- | --- | --- |
|  |  |  | **Chi-**  **square** | **Spearman**  **Correlation** | | **Chi-**  **square** | **Spearman**  **Correlation** | | **Chi-**  **square** | **Spearman**  **Correlation** | |
|  |  |  | **p-value** | **p-value** | **r** | **p-value** | **p-value** | **r** | **p-value** | **p-value** | **r** |
| **rs7521681** | 1 | VAV3 | 0.2316 | 0.8776 | -0.0057 | 0.3259 | 0.0965 | -0.1291 | 0.7432 | 0.8900 | 0.0114 |
| **rs4129267** | 1 | LOC101928101, IL6R, PSMD8P1,  SHE, TDRD10 | 0.9549 | 0.5063 | 0.0245 | 0.5740 | 0.0958 | 0.1293 | 0.1681 | 0.6264 | -0.0399 |
| **rs11902059** | 2 | COLEC11, TMSB4XP2, ALLC,  GAPDHP48, DCDC2C | 0.6943 | 0.6480 | -0.0168 | 0.8701 | 0.6660 | -0.0337 | 0.9742 | 0.8448 | -0.0163 |
| **rs17445240** | 2 | COLEC11, TMSB4XP2, ALLC,  GAPDHP48, DCDC2C | 0.4052 | 0.5789 | 0.0204 | 0.5253 | 0.4971 | -0.0529 | 0.9536 | 0.9949 | -0.0005 |
| **rs17017879** | 2 | COLEC11, TMSB4XP2, ALLC,  GAPDHP48, DCDC2C | 0.5274 | 0.6009 | 0.0192 | 0.8593 | 0.7802 | 0.0218 | 0.7868 | 0.6147 | -0.0414 |
| **rs848512** | 2 | CRIM1 | 0.2774 | 0.3010 | 0.0380 | 0.1119 | 0.0968 | -0.1313 | 0.9149 | 0.7893 | -0.0222 |
| **rs10197862** | 2 | IL1RL1, IL18R1 | **0.0253** | 0.1791 | -0.0496 | 0.5573 | 0.4499 | -0.0592 | 0.9730 | 0.7973 | 0.0214 |
| **rs9815663** | 3 |  | 0.1882 | 0.3587 | -0.0337 | 0.1676 | 0.2829 | 0.0836 | 0.8203 | 0.5713 | -0.0464 |
| **rs10044254** | 5 | FBXL7 | 0.9660 | 0.8916 | 0.0050 | 0.3769 | 0.5709 | -0.0442 | 0.5535 | 0.1498 | 0.1178 |
| **rs1837253** | 5 | TSLP, WDR36 | 0.0783 | **0.0078** | -0.0983 | 0.4926 | 0.3524 | -0.0729 | 0.2215 | 0.1710 | -0.1127 |
| **rs11745587** | 5 | C5orf56, LOC101927732, IRF1 | 0.4448 | 0.3365 | -0.0354 | 0.4377 | 0.9306 | 0.0068 | 0.2469 | 0.4186 | 0.0665 |
| **rs2549003** | 5 | C5orf56, IRF1, IL5 | 0.7214 | 0.9784 | 0.0010 | 0.8377 | 0.2492 | -0.0928 | 0.5246 | 0.1989 | 0.1085 |
| **rs6867913** | 5 | MRPL11P2, NDFIP1 | 0.8315 | 0.8343 | -0.0077 | 0.9853 | 0.7851 | 0.0215 | 0.0643 | 0.8675 | -0.0139 |
| **rs9272346** | 6 | HLA-DRB1, HLA-DQA1, HLA-DQB1 | 0.0973 | 0.3982 | -0.0310 | 0.4631 | 0.8851 | 0.0113 | 0.1267 | 0.1248 | 0.1255 |
| **rs7775228** | 6 | HLA-DQA1, HLA-DQB1, MTCO3P1 | 0.2128 | 0.1215 | 0.0568 | 0.0557 | **0.0201** | -0.1798 | 0.4612 | 0.1857 | -0.1083 |
| **rs987870** | 6 | HLA-DPA1, HLA-DPB1, RPL32P1,  HLA-DPA2, COL11A2P1, HLA-DPB2 | **0.0415** | 0.2689 | 0.0406 | 0.6751 | 0.4900 | -0.0541 | 0.4414 | 0.4165 | -0.0666 |
| **rs2473967** | 6 |  | 0.9515 | 0.8975 | 0.0048 | 0.8983 | 0.5869 | -0.0433 | 0.6981 | 0.4192 | -0.0664 |
| **rs6456042** | 6 |  | 0.3125 | 0.4525 | -0.0276 | 0.2593 | 0.7075 | -0.0292 | 0.7923 | 0.3647 | 0.0745 |
| **rs6967330** | 7 | CDHR3 | 0.4993 | 0.1775 | 0.0495 | 0.8612 | 0.3953 | 0.0662 | 0.6995 | 0.8878 | 0.0116 |
| **rs9297216** | 8 |  | 0.6153 | 0.2045 | -0.0475 | 0.5919 | 0.7932 | -0.0210 | 0.4068 | 0.0624 | 0.1568 |
| **rs1425902** | 8 | EI24P5, OPRK1 | **0.0067** | 0.4787 | -0.0264 | 0.3263 | 0.0689 | -0.1433 | 0.6839 | 0.6659 | 0.0359 |
| **rs6469488** | 8 |  | 0.5098 | 0.4342 | -0.0287 | 0.5810 | 0.3731 | 0.0694 | 0.8050 | 0.9780 | -0.0023 |
| **rs16937883** | 9 |  | 0.5219 | 0.1331 | 0.0551 | 0.5588 | 0.5491 | -0.0467 | **0.0247** | 0.9998 | 0.0000 |
| **rs2378383** | 9 | CHCHD2P9 | 0.1000 | **0.0314** | -0.0789 | 0.8415 | 0.5800 | 0.0431 | 0.3379 | 0.3193 | -0.0819 |
| **rs11252394** | 10 | LINC00702 | 0.1022 | 0.2014 | 0.0469 | 0.9243 | 0.6936 | -0.0307 | 0.4023 | 0.2258 | -0.0991 |
| **rs4752066** | 10 | LOC101927731, EMX2OS | 0.2965 | 0.6555 | 0.0164 | 0.2716 | 0.4842 | -0.0547 | 0.7088 | 0.4779 | -0.0582 |
| **rs7130588** | 11 | C11orf30 | 0.3983 | 0.9297 | 0.0032 | **0.0103** | 0.3151 | 0.0782 | 0.3658 | 0.2048 | -0.1045 |
| **rs2069408** | 12 | WIBG, DGKA, PMEL, CDK2, RAB5B,  LOC100131294, SUOX IKZF4 | 0.8197 | 0.6930 | -0.0145 | 0.4549 | 0.4721 | -0.0560 | 0.1792 | 0.1468 | -0.1187 |
| **rs11835157** | 12 | ELK3, CDK17 | 0.0888 | **0.0385** | -0.0762 | 0.5547 | 0.7682 | -0.0233 | 0.7792 | 0.5247 | -0.0527 |
| **rs9319321** | 13 |  | 0.6139 | 0.9694 | 0.0014 | 0.8856 | 0.5045 | 0.0525 | **0.0448** | 0.6503 | -0.0373 |
| **rs1323555** | 13 | CYSLTR2 | 0.3030 | 0.4577 | 0.0273 | 0.9435 | 0.5374 | -0.0481 | 0.3261 | 0.0523 | 0.1582 |
| **rs1930336** | 13 |  | **0.0103** | 0.9408 | -0.0027 | 0.6775 | 0.9838 | -0.0016 | 0.2575 | 0.3940 | -0.0701 |
| **rs744910** | 15 | SMAD3, AAGAB | 0.2580 | 0.1565 | -0.0520 | 0.5305 | 0.4165 | -0.0633 | 0.3876 | 0.5711 | 0.0465 |
| **rs17294280** | 15 | SMAD3, AAGAB | 0.2869 | 0.1484 | 0.0530 | 0.2484 | 0.7206 | 0.0279 | 0.7864 | 0.4976 | -0.0556 |
| **rs2388639** | 16 |  | 0.9136 | 0.6844 | -0.0150 | 0.6153 | 0.7075 | -0.0293 | 0.6454 | 0.6554 | 0.0369 |
| **rs6563898** | 16 | CDH13 | 0.9416 | 0.8542 | 0.0068 | 0.7476 | 0.3860 | -0.0681 | 0.5315 | 0.3188 | 0.0822 |
| **rs10521233** | 17 |  | 0.1265 | 0.7787 | 0.0104 | 0.7664 | 0.7136 | 0.0290 | 0.5664 | 0.5842 | -0.0460 |
| **rs11078927** | 17 | IKZF3, ZPBP2, GSDMB, ORMDL3,  LOC101928947, LRRC3C | 0.8467 | 0.7961 | -0.0095 | 0.1403 | 0.3878 | 0.0673 | 0.0965 | 0.3473 | -0.0770 |
| **rs6503525** | 17 | GSDMB, ORMDL3, LOC101928947,  LRRC3C, GSDMA, PSMD3 | 0.6030 | 0.4347 | -0.0292 | 0.0921 | 0.1174 | -0.1239 | **0.0297** | 0.0805 | -0.1472 |
| **rs10411428** | 19 | LOC100419835, ZNF841, ZNF616,  RPL37P23 | 0.1544 | 0.2914 | -0.0388 | 0.8416 | 0.4517 | 0.0586 | **0.0028** | **0.0008** | 0.2717 |
| **rs16984547** | 19 | ZNF415, ZNF347, ZNF665, ZNF818P,  LOC100419845, NDUFV2P1 | 0.1450 | 0.8673 | 0.0062 | 0.7620 | 0.2062 | 0.0983 | 0.7221 | 0.8680 | -0.0136 |
| **rs4815617** | 20 | CDC25B, LOC101929125,  AP5S1, MAVS, PANK2 | 0.9669 | 0.9996 | 0.0000 | **0.0307** | 0.5355 | -0.0484 | 0.2680 | 0.3164 | 0.0823 |
| **rs2284033** | 22 | TMPRSS6, IL2RB, C1QTNF6 | 0.6148 | 0.9330 | 0.0031 | 0.1111 | 0.2104 | 0.0974 | 0.7021 | 0.7216 | 0.0292 |

Reference

[1] Providing large-scale and comprehensive annotations on human genomic variants in the next generation sequencing era. Cheng, Y.C., Hsiao, F.C., Yeh, E.C., Lin, W.J., Tang, Louis, Tseng, H.C., Wu, H.T., Liu, C.K., Chen, C.C., Chen, Y.T., and Yao, Adam (2012) VarioWatch: providing large-scale and comprehensive annotations on human genomic variants in the next generation sequencing era doi:10.1093/nar/gks397
